# Exposure to per- and polyfluoroalkyl substances (PFAS) and type 2 diabetes risk

**DOI:** 10.3389/fendo.2022.965384

**Published:** 2022-08-05

**Authors:** Katherine Roth, Michael C. Petriello

**Affiliations:** ^1^ Institute of Environmental Health Sciences, Wayne State University, Detroit, MI, United States; ^2^ Department of Pharmacology, School of Medicine, Wayne State University, Detroit, MI, United States

**Keywords:** PFAS toxicity, Per- and polyfluoroalkyl substances, diabetes, cardiometabolic disease, hyperglycemia, insulin

## Abstract

Per- and polyfluoroalkyl substances (PFAS) are ubiquitous man-made chemicals found in consumer products including fabrics, food packaging, non-stick coatings, and aqueous film-forming foams. PFAS are stable and extremely resistant to degradation, resulting in high persistence throughout the environment as well as in human blood. PFAS consist of a large family of synthetic chemicals, with over 4000 distinct varieties having been identified and around 250 currently being manufactured at globally relevant levels. Numerous epidemiological studies have linked exposure to PFAS with adverse health effects ranging from immunotoxicity, cardiometabolic disease, developmental and reproductive effects, cancer, and recently type 2 diabetes. Several studies have demonstrated associations between serum PFAS concentrations and glycemic indicators of type 2 diabetes including glucose, insulin, and HOMA-IR in adolescent and adult cohorts. In addition, some studies have shown positive associations with incident type 2 diabetes and multiple PFAS. However, the link between PFAS exposure and the development of diabetes continues to be a disputed area of study, with conflicting data having been reported from various epidemiological studies. In this mini review we will summarize the current state of the literature linking PFAS to type 2 diabetes and discuss important future directions including the use of more complex mixtures-based statistical analyses.

## Introduction

Diabetes is a condition affecting 537 million adults worldwide (1 in 10) and is predicted to rise by over another hundred million by 2030, with type 2 diabetes making up over 90% of cases (1). Diabetes has set a high burden on both individuals and health care systems, with global health expenditure due to diabetes in 2021 reaching over $966 billion (1). Diabetes is a chronic, metabolic condition in which individuals have elevated blood glucose levels, also known as hyperglycemia. When left untreated, hyperglycemia can lead to serious damage to blood vessels, nerves, and various tissues and organs, especially the heart and kidneys (2). In the United States, diabetes is the seventh leading cause of death (3). Much of this is due to diabetes being a major risk factor for the development of cardiovascular disease (CVD), which is the most common cause of death in those with Type 2 diabetes (T2D), accounting for over 52% (4, 5). Diabetes occurs when the body is not able to produce enough insulin (T1D) or when the body cannot effectively use the insulin that is being produced (T2D) (6). Increases in blood glucose leads to increased synthesis and secretion of insulin by beta cells in the pancreas. Insulin is an endocrine peptide hormone that binds to receptors in target cells and activates signaling pathways resulting in various metabolic changes, most notably increased glucose uptake (7). T2D is the most common type of diabetes and occurs when your pancreas does not produce enough insulin and cells become resistant to insulin, leading to lower cellular uptake of glucose and therefore higher blood glucose levels (3, 6). Diabetes is commonly diagnosed by measuring glucose and/or insulin after an oral glucose tolerance test or by hemoglobin A1C test (8). Insulin resistance, another hallmark of diabetes, can be measured by calculating the homeostatic model assessment (HOMA-IR) (9). Long-standing studies have demonstrated how genetic and lifestyle factors have a major impact on the development and progression of T2D (10–12). Several genes involved in beta-cell development or function have been identified through genome-wide association studies that confer T2D risk (11, 12). In addition, little physical activity, a poor diet, smoking, stress, and high body mass index have all been attributed in the promotion of T2D prevalence and incidence (10). However, studies have begun to investigate additional environmental factors that potentially impact on the development of diabetes, such as exposure to environmental contaminants acting as endocrine-disrupting chemicals, including per- and polyfluoroalkyl substances (13–15).

Per- and polyfluoroalkyl substances (PFAS) are man-made chemicals containing a fluorinated carbon chain that act as persistent organic pollutants (POPs) (16). These chemicals have been manufactured and utilized for decades in countless consumer and industrial products for their excellent surfactant properties (17, 18), including fabrics, food packaging, non-stick coatings, and aqueous film-forming foams (16, 19–21). PFAS as a class of chemicals may represent upwards of 4000 distinct varieties of which 250 are currently being manufactured at globally relevant levels (22, 23). PFAS are very stable and extremely resistant to degradation, resulting in high persistence throughout the environment, including water sources, soil, plants, animals, as well as humans around the globe (24–27). Humans are exposed to PFAS *via* several exposure routes, the principal ones being through contaminated drinking water or food supplies (26, 28). Even among the general population, PFAS can be detected in the blood, with data from the National Health and Nutrition Examination Survey (NHANES) 2003-2004 reporting that PFAS can be measured in 98% of U.S. adults (29–31). PFOS and PFOA were measured in serum at 20.7 ug/L and 3.9 ug/L averaged for the whole population, respectively (29). Although legacy halogenated POPs, such as PCBs, have been linked to diabetes risk for years (32), their levels in the environment and serum have steadily decreased (32, 33). Thus, it is important to investigate associations between halogenated POPs of emerging concern, such as PFAS, and the risk of T2D.

## PFAS and glucose homeostasis

Since PFAS are persistent organic pollutants ubiquitous throughout the environment, bioaccumulating in humans across the globe, research exploring the adverse health effects associated with exposure to PFAS has greatly increased over the past few decades (34, 35). PFAS have been associated with multiple chronic diseases in epidemiological studies including immunotoxicity, cardiometabolic disease, developmental and reproductive effects, and cancer (36–40). More recent studies have begun to explore associations between PFAS exposure and diabetes (14, 15). However, the link between PFAS exposure and the development of diabetes continues to be a disputed area of study, with conflicting data having been reported from various epidemiological studies. **Table 1** outlines many of these epidemiological studies examining the association of PFAS exposure with the development of T2D. Of these, several studies have demonstrated positive associations between serum PFAS concentrations and an increase in glycemic indicators of T2D. In 2016, Domazet et al. analyzed data from the European Youth Heart Study, which collected samples from participants from 1997-2009 during childhood (9 years), adolescence (15 years) and young adulthood (21 years) (41). PFOA exposure in childhood was found to be associated with decreased β-cell function at 15 years of age (HOMA-β: ~ -12% decrease change in outcome by 10 ng/ml increase in PFOA exposure). In 2016, Kim et al. published results from a double-blind, placebo-controlled study with elderly (60+ years) Korean subjects recruited in 2011 that were given vitamin C treatments for 4 weeks (42). Of the 8 PFAS measured, the authors found PFOS and PFDoDA to be positively associated with HOMA-IR at baseline and after placebo treatment (β_PFOS_ = 0.60 increase by IQR; 95% CI: -0.002, 1.19, p=0.053; β_PFDoDA_ = 0.83; 95 % CI 0.23, 1.44, p=0.01). Interestingly, these effects were not observed after vitamin C treatment. In 2017, Cardenas et al. examined associations between plasma PFAS concentrations and glycemic indicators from participants of the Diabetes Prevention Program (DPP), who were recruited between 1996-99 (43). At baseline, a doubling in PFOS and PFOA concentrations was associated with increased HOMA-IR (β_PFOS_ = 0.39; 95% CI: 0.13, 0.66; β_PFOA_ = 0.64; 95% CI: 0.34, 0.94], increased HOMA-β (β_PFOS_ = 9.62; 95% CI: 1.55, 17.70; β_PFOA_ = 15.93; 95% CI: 6.78, 25.08), increased fasting proinsulin (β_PFOS_ = 1.37 pM; 95% CI: 0.50, 2.25; β_PFOA_ = 1.71 pM; 95% CI: 0.72, 2.71), and increased HbA1c (β_PFOS_ = 0.03%; 95% CI: 0.002, 0.07; β_PFOA_ = 0.04%; 95% CI: 0.001, 0.07). However, no strong associations were found during the follow-up period for PFAS and the above diabetes indicators. In 2019, a study was published that analyzed serum samples from a cohort in Tianjin, China ranging in age from 19 to 87 years old that had been recruited in 2017 (44). The authors found that a 1% increase in PFOA and PFNA had significant positive associations with fasting glucose levels: 0.018% [(95% CI): 0.004%, 0.033%] and 0.022% (95% CI: 0.007%, 0.037%), respectively. In addition, a 1% increase in PFNA, PFHxA, and PFHxS were had a significant positive association with HbA1c levels: 0.018% (95% CI: 0.003%, 0.033%), 0.030% (95% CI: 0.010%, 0.051%), 0.007% (95% CI: 0.003%, 0.011%), respectively. In 2019, Alderete et al. published an analysis of a cohort of overweight and obese Hispanic children (8-14 years) recruited from 2001-2012 from Los Angeles, California (45). A 2-hour OGTT was performed at baseline and at a follow-up 1-3 years later. The study found that each ln(ng/ml) increase in PFOA and PFHxS was significantly associated with an increase in 2-hour glucose levels (PFOA: 30.6 mg/dL (95% CI: 8.8–52.4) and PFHxS: 10.2 mg/dL (95% CI: 2.7–17.7)). In 2021, Zeeshan et al. analyzed data from the “Isomers of C8 Health Project in China,” which collected samples from participants 35+ years old between 2015-2016 (46). The levels of several PFAS were measured, including PFOA, PFOS, PFHxS, PFHpS, PFNA, PFDA, PFUnDA, and PFTrDA. For isomers of PFOS, PFOA, PFHxS, PFHpS, PFNA, PFDA, and PFUnDA significant positive associations were found with fasting blood glucose, fasting insulin, and HOMA-IR. For example, an increase in branched PFOS was determined to be positively associated with fasting blood glucose levels (β_PFOS_ = 0.25, 95% CI: 0.18, 0.33), fasting insulin (β_PFOS_ = 2.19, 95% CI: 1.44, 2.93) and HOMA-IR (β_PFOS_ = 0.69, 95% CI: 0.50, 0.89). In 2021, Valvi et al. analyzed data from a prospective cohort made up of those born in the Faroes Islands (born 1986-87) (47). Blood was collected and PFAS measured in cord whole blood and at ages 7, 14, 22, and 28 years. PFOS exposure was associated with a decrease in insulin sensitivity and an increase in insulin secretion. For example, the log-ISI per PFOS doubling was: β_PFOS_ = 0.12 (95%CI: 0.02, 0.22), for prenatal exposures. Although associations for PFOA and PFHxS were often in the same direction as PFOS, they were not statistically significant. In addition, the overall life-course of PFOS exposure was found to be associated with altered glucose homeostasis (p=0.04).

**Table 1:** Key physiological endpoints from studies that show positive associations between PFAS exposure and markers of T2D risk.

Citation	Cohort	PFAS Serum Concentrations		Physiological Endpoints
Domazet et al., 2016(41)	European Youth Heart Study (1997-2009)	9-year Median PFOA (ng/mL): • Male = 9.7 • Female = 9.0		↓ Decreased HOMA-β (at 15 yrs) significantly associated with PFOA (at 9 yrs)
Kim et al., 2016 (42)	Vitamin C intervention study in the elderly (60+ years old) in Seoul, Korea (2011-2012)	Mean (ng/mL): • PFOS = 10.04 • PFOA = 4.61 • PFDoDA = 0.25		↑ Increased HOMA-IR significantly associated with PFOS and PFDoDA
Cardenas et al., 2017 (43)	Diabetes Prevention Program (1996-1999)	Geometric mean (ng/mL): • PFOA = 4.82 • PFOS = 26.38		↑ Increased HOMA-IR significantly associated with PFOS and PFOA ↑ Increased HbA1c significantly associated with PFOS and PFOA
Duan et al., 2020 (44)	Workers at Nankai University, Tianjin, China (19-87 years old) (2017)	Geometric mean (ng/ml): • PFOA = 14.82 • PFNA = 3.14 • PFHxS = 0.06 • PFHxA = 0.54		↑ Increased fasting glucose significantly associated with PFOA and PFNA ↑ Increased HbA1c significantly associated with PFNA, PFHxS, and PFHxA
Alderete et al., 2019 (45)	Study of Latino Adolescents at Risk of T2D (Overweight and obese Hispanic children 8-14 yrs old) (2001-12)	Geometric mean (ng/mL): • PFOA = 2.78 • PFOS = 12.22 • PFHxS = 1.65		↑ Increased 2-hour glucose levels (GTT) significantly associated with PFOA and PFHxS
Zeeshan et al., 2021 (46)	Isomers of C8 Health Project in China (35+ years old) (2015-2016)	Median (ng/mL): • n-PFOA = 4.63 • n-PFOS = 9.95 • br-PFOS = 14.25 • n-PFHxS = 2.56	• br-PFHxS = 0.01 • PFHpS = 0.98 • PFNA = 1.44 • PFDA = 0.60 • PFUnDA = 0.64	↑ Increased fasting glucose significantly associated with br-PFOS, n-PFOS, br-PFHxS, n-PFHxS, n-PFOA, PFHpS, PFNA, PFDA, and PFUnDA ↑ Increased HOMA-IR significantly associated with br-PFOS, n-PFOS, br-PFHxS, n-PFHxS, n-PFOA, PFHpS, PFNA, PFDA, and PFUnDA
Valvi et al., 2021 (47)	Participants born in the Faroes Islands during 1986-87 (1986-2015)	Median cord blood (ng/mL): • PFOS = 2.92 • PFOA = 0.54 7-year serum (ng/mL): • PFOS = 31.5• PFOA = 5.06		↓ Decreased insulin sensitivity significantly associated with PFOS ↑ Increased β-cell function significantly associated with PFOS

Down arrow = decreased. Up arrow = increased.

Although several studies demonstrated a positive association between serum PFAS levels and an increase in diabetes indicators, various studies have also determined there to be a non-significant or inverse association, as outlined in **Table 2**. In 2018, Liu et al. examined subjects from the NHANES (2013-14) to analyze associations between isomers of PFOA/PFOS and a wide-ranging biochemistry profile (48). They found that branched PFOA and linear PFOS were significantly associated with decreased fasting glucose (β_br-PFOA_ = -4.07 ± 1.69mg/dL(SE))(β_n-PFOS_ = -1.8 ± 0.72mg/dL). No significant associations with PFAS were found for 2hr glucose (GTT), insulin levels, or HOMA-IR. All forms of PFOA were found to be associated with decreased HbA1c (β_PFOA_ = -0.12 ± 0.05). Interestingly, β-cell function, measured by HOMA2-IR, was found to be significantly increased by n-PFOA (βn-_PFOA_ = 0.12 ± 0.05). In 2010, Nelson et al. analyzed data from NHANES (2003-04) for associations between PFAS (PFOA, PFNA, PFOS, and PFHxS) and insulin resistance, but found no significant association between the PFAS examined and HOMA-IR (49). In 2012, Fisher et al. analyzed data from the Canadian Health Measures Survey (2007-09) for associations between PFOA, PFOS, and PFHxS levels and plasma glucose, plasma insulin, and HOMA-IR (50). No association was found between the PFAS examined and these diabetes indicators. In 2017, Fleisch et al. analyzed blood samples from children (~8 years) in Project Viva, a cohort from the Boston, Massachusetts area that had samples collected from 1999-2002 for prenatal visits and 2007-2010 for child plasma (51). Children that had higher concentrations of PFOA, PFOS, and PFDeA were associated with lower HOMA-IR per interquartile range (PFOA: -10.1% (95% CI: -17.3, -2.3), PFOS: -10.1% (-16.4, -3.3), PFDeA: -14.7% (-22.1, -6.5)). Sex-specific patterns were also observed between childhood PFAS concentrations and HOMA-IR, with childhood PFOA, PFOS, and PFDeA levels associated with decreased HOMA-IR in females but not significantly associated in males. In 2019, Fassler et al. published a cross-sectional study of young girls (6-8 years) from the Cincinnati area recruited from 2004-2006 (52). While no significant associations were found between PFAS and fasting insulin and glucose, PFOA levels were found to have a borderline inverse statistical association with fasting insulin levels (p=0.09), insulin resistance (HOMA-IR) (p=0.09), and a positive borderline effect on insulin sensitivity (ISI) (p=0.10). Many of the studies demonstrating a negative or non-significant association between PFAS exposure and glucose/insulin levels and resistance analyzed national health surveys with lower risk populations and/or lower PFAS plasma concentrations. For example, the NHANES (2003-2004) measured a median PFOA serum concentration of 3.8 ng/mL and the Canadian Health Measures Survey (2007-2009) measured a median PFOA concentration of 2.46 ng/mL. The study by Fassler et al., examining children in the Cincinnati area, had the highest media PFOA concentration of 7.7 ng/mL, which could be a reason for their borderline positive associations. Of the studies demonstrating significant positive correlations, cohorts were often at higher risk, including the elderly or obese children, or had a higher median PFOA concentration from 4.6-14.82 ng/mL.

**Table 2:** Key physiological endpoints from studies that show non-significant or inverse associations between PFAS exposure and markers of T2D risk.

Citation	Cohort	PFAS Serum Concentrations	Physiological Endpoints
Liu et al., 2018 (48)	NHANES (2013-2014)	Geometric mean PFOA (ng/mL): • Total PFOA = 1.86 • n-PFOA = 1.75 • br-PFOA = 0.08 Geometric mean PFOS (ng/mL): • Total PFOS = 5.28 • n-PFOS = 3.70 • br-PFOS = 1.39	↓Decreased HbA1c significantly associated with PFOA No significant associations between PFAS examined and 2hr glucose (GTT), insulin levels, or HOMA-IR
Nelson et al., 2010 (49)	NHANES (2003-2004)	Median (ng/mL): • PFOA = 3.9 • PFOS = 21.0 • PFHxS = 1.8	No significant associations between PFAS examined and HOMA-IR.
Fisher et al., 2013 (50)	Canadian Health Measures Survey (2007-2009)	Geometric mean PFOA (ng/mL): • Male = 2.92 • Female = 2.18 Geometric mean PFOS (ng/mL): • Male = 11.0 Female = 6.99	No significant associations between PFAS examined and fasting insulin, fasting glucose, and HOMA-IR.
Fleisch, 2017 (51)	Mother-child pairs in Project Viva (mothers recruited 1999-2002, mid-childhood samples from 2007-2010)	Mid-childhood PFAS concentrations (Geometric mean, ng/mL): • PFOA: 4.2 • PFOS: 6.2 • PFDeA: 0.3	↓Decreased HOMA-IR significantly associated with mid-childhood concentrations of PFOA, PFOS, and PFDeA Sex-stratified analysis showed PFOA, PFOS, and PFDeA concentrations inversely associated with HOMA-IR in females only
Fassler et al., 2019 (52)	Participants were 6 to 8-year old girls from Greater Cincinnati (2004-2006)	Median (ng/mL): • PFOA: 7.30 • PFOS: 13.60 • Me-PFOSA-AcOH: 0.80 • PFNA: 1.40• PFHxS: 5.20	Borderline inverse association between insulin resistance and both PFOA and Me-PFOSA-AcOH Borderline direct association between insulin sensitivity and both PFOA and Me-PFOSA-AcOH

Down arrow = decreased.

## PFAS and T2D

Furthermore, there have also been many studies performed investigating the association between exposure to PFAS and incidence of T2D, although associations often varied depending on the specific PFAS examined. In 2009, Lundin et al. published data analyzing a highly exposed cohort of employees from a 3M company in Cottage Grove, Minnesota that produced PFOA employed between 1947 and 1997 (53). The authors reported an increased risk of diabetes and diabetes-related deaths in workers with moderate exposure (HR = 3.7; 95% CI: 1.4-10.1), but not in low or high exposures. A 2018 study by Sun et al. examined the association between PFAS exposure and T2D in the Nurses’ Health Study II (NHSII), a prospective cohort of female nurses in the U.S. enrolled who provided blood samples in 1995-2000 (54). Higher plasma concentrations of PFOS and PFOA were positively associated with increased risk of T2D. For PFOA and PFOS, respectively, comparing first and third tertiles, OR_PFOS_ = 1.62 (95% CI: 1.09, 2.41; p_trend_ = 0.02) and OR_PFOA_ = 1.54 (95% CI: 1.04, 2.28; p_trend_ = 0.03). However, plasma concentrations of PFHxS, PFNA, and PFDA did not show association with T2D. As mentioned previously, Zeeshan et al. examined data from Chinese adults from the “Isomers of C8 Health Project in China (46).” In addition to finding positive associations between PFAS and several diabetes indicators, they also found that serum concentrations of all PFAS were associated with increased risk of T2D in adults. Branched PFOS had the highest risk (OR=5.41, 95% CI: 3.68–7.96). The T2D incidence associations were generally stronger in women compared to men. In 2018, He et al. examined cross-sectional data from NHANES (2003–12) in order to examine the association of serum PFAS levels with diabetes prevalence (55). It was found that PFOA levels were strongly associated with diabetes prevalence in men (OR: 2.66, 95% CI: 1.63–4.35; p for trend = 0.001), but not women. Furthermore, PFOS, PFHxS, and PFNA were not found to be associated with diabetes prevalence regardless of gender. In 2019, Girardi et al. published an analysis of the association of PFAS exposure and mortality risk in a cohort of workers for a factory that produced PFOA and PFOS compared to workers from a metalworking factory from 1960-2008 (56). The authors found an increased relative risk for mortality from diabetes consequences (RR: 5.95; CI: 1.08–32.8) from workers in the PFAS factory compared to the metalworking factory. In 2017, as mentioned previously, Cardenas et al. examined participants of the Diabetes Prevention Program (DPP) (43). Although the authors found several PFAS associated with increased insulin resistance (HOMA-IR) and HbA1c levels, they did not find plasma PFAS concentrations to be strongly associated with incidence of T2D. However, the same group performed another study in 2019, including results from the Diabetes Prevention Program Outcomes Study (DPPOS), which was a 15-year follow-up study from the DPP (57). A doubling of PFOA was positively associated with a 14% increase in diabetes risk (HR 1.14, 95% CI 1.04, 1.25). However, this association was not observed in the lifestyle intervention group.

Although several studies demonstrated a positive association between exposure to PFAS and risk of T2D, various studies have also determined there to be a non-significant or inverse association between exposure to PFAS and risk of T2D. In 2019, Donat-Vargas et al. examined participants from the Swedish Vasterbotten Intervention Programme (VIP) prospective cohort, a sub-cohort of the Northern Sweden Health and Disease Study begun in 1985 (58). When comparing the first and third tertile, the odds ratio of T2D for the sum of PFAS was 0.52 (95% CI: 0.20, 1.36). Overall, PFAS was determined to be weakly inversely associated with the risk of future T2D, but mostly non-significant. Multiple groups have also analyzed the effects of PFAS exposure on T2D risk in an occupational cohort from the C8 Health Project highly exposed to PFOA, which found either no significant association between PFOA exposure and incidence of T2D (59, 60) or that PFAS serum levels were negatively associated with T2D: PFHxS (OR (CI))= 0.74 (0.71–0.77), PFOA (OR (CI))= 0.87 (0.89–0.91), PFOS (OR (CI))= 0.86 (0.82–0.90), and PFNA (OR (CI))= 0.94 (0.88–1.00) (61). However, these studies are limited by the cross-sectional nature and the fact that validation of T2D was only done for those who self-reported diabetes. In 2020, a study by Charles et al. analyzed samples from women in the Norwegian Women and Cancer study (62). Repeated measures of blood samples were collected prior to T2D diagnosis (2001/02) and then after T2D diagnosis (2005/6) as well as from non-diabetic controls. This study found no significantly increased OR for T2D incidence or prevalence with PFAS exposure. A recent study by Schillemans et al. published in 2021 examined how PFAS-related metabolites associate with risk of T2D in the Swedish VIP prospective cohort (63). PFAS levels were found to correlate positively with both glycerophospholipids and diacylglycerols. However, glycerophospholipids were found to associate inversely with risk of T2D, while diacylglycerols correlated positively with risk of T2D. These findings may play a role in understanding the conflicting results found for the association of T2D and PFAS levels in some epidemiological studies. In 2021, Han. et al. examined participants in a case-control study from Shandong Province, East China, recruited from 2016-2017 (64). The authors found that exposure to PFOA, PFOS, PFDA, PFUnDA, PFHxS, and 6:2 Cl-PFESA were all inversely associated with risk of T2D with significant ORs <1. However, serum PFOA was positively associated with fasting plasma glucose levels (β = 0.04, 95% CI: 0.0003, 0.08), which may promote T2D.

Although many studies have shown positive associations between PFAS exposure and T2D risk, others have shown inverse or no associations which may be due to exposure levels in a particular population or non-monotonic dose responses (65, 66). In 2018, Mancini and colleagues analyzed the link between T2D and dietary exposure to PFOA and PFOS in the E3N (Etude Epidémiologique auprès de femmes de l’Education Nationale) prospective cohort, which includes women in France between 40-60 years old and 15 years of follow-up (65). The mean dietary exposure was estimated to be 0.50ng/kg/day PFOS and 0.86ng/kg/day PFOA. This study found PFOA to have an inverse U-shape association with T2D, with the strongest positive effect on non-obese women. PFOS had a positive nonlinear association with T2D only among the non-obese women. In 2021, Duan et al. investigated the associations of various PFASs to T2D risk over a range of exposure levels (66). They examined data from participants in a case-control study, with half having confirmed T2D and half controls. They found that serum PFHxS and PFHpA displayed an inverted U-shaped dose-response, highest significant positive association with T2D risk in the middle tertiles. However, most other serum PFAS demonstrated a negative association with T2D risk, especially at higher levels.

## Future directions

Analysis of PFAS in epidemiological studies have uncovered important associations between single PFAS and diabetes risk factors and incidence, and it will continue to be useful for emerging PFAS. However, this approach lacks the environmental relevance that comes with evaluating more realistic environmental mixtures. It will be important in the future to advance these findings by examining PFAS mixtures and incorporating the use of mixture statistics. Mixtures can interact to produce combination effects other than those predicted by individual constituent chemicals, including interactions leading to additive or synergistic results that promote significant, measurable effects. A couple recent studies have begun to employ mixture statistics in their approach to estimating the combined effects of PFAS mixtures on diabetes incidence and estimated glomerular filtration rates. In 2021, Lin et al. published a follow-up study examining participants in the Diabetes Prevention Program trial (DPP, 1996–2002) and the later Outcomes Study (DPPOS, 2002–2014) (67). The authors evaluated the associations between PFAS levels and repeated measures of eGFR, individually, for PFOA, PFOS, PFHxS, EtFOSAA, MeFOSAA, and PFNA. It was found that, individually, total PFOS was observed to be inversely associated with eGFR: each doubling of total plasma PFOS was associated with -1.50 mL/min/1.73m ^2^ lower eGFR (95% CI: −2.81, −0.19). Quantile-based g-computation was then used to estimate the effects of the six PFAS as a mixture. As a mixture, it was found that each quartile increase in concentration of the PFAS mixture was associated with -2.26 mL/min/1.73 m ^2^ lower eGFR (95% CI: −4.12, −0.39) at DPPOS Year 5, ~9 years since the PFAS measurements. Furthermore, in 2022, Park et al. published a study examining the association of PFAS with incident diabetes in the Study of Women’s Health Across the Nation Multi-Pollutant Study (SWAN-MPS) (1999-2017) (68). The authors evaluated the associations between PFAS levels and diabetes incidence, individually, and found that the HR (95% CI) comparing the first and third tertile was 1.67 (1.21, 2.31) for n-PFOA (p_trend_ = 0.001), 1.58 (1.13, 2.21) for PFHxS (p_trend_ = 0.003), 1.36 (0.97, 1.90) for Sm-PFOS (p_trend_ = 0.05), and 1.85 (1.28, 2.67) for MeFOSAA (p_trend_ = 0.0004). Quantile-based g-computation was then used to evaluate the combined effects of seven PFAS as a mixture (n-PFOA, PFNA, PFHxS, n-PFOS, Sm-PFOS, MeFOSAA, and EtFOSAA). Exposure to the PFAS mixture was associated with an HR of 2.62 (95% CI 1.12, 6.20). As PFAS exist as complex mixtures in real-life settings, it will be important in the future to consider the impacts of PFAS as a complex mixture to our health. Furthermore, although in this review we have explored the effects of PFAS on T2D incidence and risk factors, the mechanisms potentially involved in these effects remain largely unknown.

Animal models have been used as a method for uncovering underlying mechanisms related to the effects of PFAS on glucose and insulin metabolism, as outlined in **Table 3**. A couple studies performed in rodent models demonstrated contrasting conclusions. In both, mice were exposed to PFOA at a dose of 1.25 mg/kg/day for 28 days. In 2015, Yan et al., PFOA exposure in mice was associated with increased insulin sensitivity and increased activation of the PI3K-AKT signaling, which is downstream of insulin receptor signaling (69). In 2017, Zheng et al. also exposed mice to PFOA for 28 days and observed that PFOA exposure increased blood glucose and decreased glycogen and glucose in the liver, while insulin levels in the blood were not significantly affected (70). Recently, *in vitro* studies have also been used to investigate mechanisms related to the effects of PFAS on glucose and insulin metabolism and β-cell function (73). In addition to their rodent model, Qin et al. exposed pancreatic β-cells to PFOS, which decreased their insulin release capacity following glucose stimulation (71). When the authors also exposed mice to PFOS for 28 days, pancreas weight and islet size were decreased. Duan et al. also demonstrated that exposure of pancreatic β-cells to PFOS resulted in impaired insulin secretion, possibly through inhibition of SIRT1 activity and decreased mitochondrial ATP production and Ca^2+^ influx (72). Further studies examining the effects of PFAS exposure on β-cell function and modulation of signaling pathways are needed in order to gain a better understanding of the mechanisms linking PFAS exposure to diabetes.

**Table 3:** Key mechanistic endpoints from animal and *in vitro* PFAS exposure studies related to glucose and insulin metabolism.

Citation	Experimental Model	PFAS Serum Concentrations or *in vitro* Exposures	Physiological Endpoints
Yan et al., 2015 (69)	Male Balb/c mice (age 6-8 weeks) were administered PFOA diluted in water for 28 days at the following doses: 0, 0.08, 0.31, 1.25, 5, and 20 mg/kg/d *via* oral gavage.	Mean Serum PFOA: • 0.08 mg/kg/d – 2.24 ug/mL • 0.31 mg/kg/d – 8 ug/mL • 1.25 mg/kg/d – 25 ug/mL • 5 mg/kg/d – 55 ug/mL • 20 mg/kg/d – 105.3 ug/mL	↑ Increased insulin and glucose sensitivity (GTT and ITT tests). ↑ Increased activation of PI3K-AKT signaling pathway.
Zheng et al., 2017 (70)	Male Balb/c mice (age 6-8 weeks) were administered 1.25 mg/kg/d PFOA for 28 days.	Mean PFOA (± SE): • 55.5 ± 0.50 μg/mL (serum) • 125.9 ± 10.0 μg/g (liver)	↑ Increased fasting blood glucose ↓ Decreased glycogen and glucose content in the liver. No significant change in blood insulin and insulin sensitivity
Qin et al., 2022 (71)	*In vivo:* C57BL/6 mice administered 1-10 mg/kg/day PFOS *via* oral gavage for 28 days. *In vitro:* Mouse β-TC-6 pancreatic cells	Mean Serum PFOS in mice (± SE): • 0 mg/kg/d – 3 ± 2 μmol/L • 1 mg/kg/d – 63 ± 9 μmol/L • 5 mg/kg/d – 234 ± 27 μmol/L • 10 mg/kg/d – 348 ± 47 μmol/Lβ-TC-6 cells: 48-hour exposure to PFOS at 50-100 μmol/L	↓ PFOS exposure in male mice caused reductions of pancreas weight and islet size. ↓ PFOS exposure decreased the insulin release capacity of β-TC-6 cells after glucose stimulation.
Duan et al., 2021(72)	Mouse β-TC-6 pancreatic cells.	β-TC-6 cells: exposure to PFOS at 0-200 µM for 24 or 48 hours.	↓ PFOS exposure (48 h) impaired glucose stimulated insulin secretion. PFOS exposure decreased mitochondrial glucose-induced ATP production and Ca^2+^ influx.

Down arrow = decreased. Up arrow = increased.

## Conclusions

Overall, while studies have long been shown to demonstrate the major impact of genetic and lifestyle factors on the development and progression of T2D (10–12), emerging studies examined in this review have begun to investigate the impact of exposure to PFAS on glucose and insulin homeostasis as well as T2D incidence. In general, there seems to be strong evidence supporting a link between PFAS exposure and increased glucose levels and insulin resistance in individuals with multiple risk factors for diabetes, while PFAS exposures found in national surveys do not demonstrate consistent positive associations. In most cases however, these associations have been shown to vary depending on the specific PFAS examined and the gender of the subject.

The association of PFAS exposure with T2D incidence remains convoluted. It is possible these studies have produced such varied results due to an inverse U-shaped association between PFAS exposure and T2D incidence, as a couple studies have demonstrated (67, 68). Continued investigations into the relationships between PFAS exposure and T2D need to be done in order to better understand the impacts of environmental pollutants on diabetes progression and prevalence.

## Author contributions

Conceptualization, MP; Writing—Original Draft Preparation, MP and KR; Writing—Review & Editing, MP and KR. All authors have read and agreed to the submitted version of the manuscript.

## Funding

This research was supported in part by the National Institute of Environmental Health Sciences [P30ES020957, R00ES028734] and the Office of the Vice President for Research at Wayne State University. The content is solely the responsibility of the authors and does not necessarily represent the official views of the NIH.

## Conflict of interest

The authors declare that the research was conducted in the absence of any commercial or financial relationships that could be construed as a potential conflict of interest.

## Publisher’s note

All claims expressed in this article are solely those of the authors and do not necessarily represent those of their affiliated organizations, or those of the publisher, the editors and the reviewers. Any product that may be evaluated in this article, or claim that may be made by its manufacturer, is not guaranteed or endorsed by the publisher.

## References

[1] IDF Diabetes Atlas. International diabetes federation (2021). Available at: http://www.diabetesatlas.org.

[2] Brownlee M. Biochemistry and molecular cell biology of diabetic complications. Nature (2001) 414:813–20. doi: 10.1038/414813a 11742414

[3] (CDC). CfDCaP: What is diabetes? (2021). Available at: https://www.cdc.gov/diabetes/basics/diabetes.html.

[4] Benjamin EJ Virani SS Callaway CW Chamberlain AM Chang AR Cheng S. Heart disease and stroke statistics-2018 update: A report from the American heart association. Circulation (2018) 137:e67–e492. doi: 10.1161/CIR.0000000000000558 29386200

[5] Morrish NJ Wang SL Stevens LK Fuller JH Keen H. Mortality and causes of death in the WHO multinational study of vascular disease in diabetes. Diabetologia (2001) 44 Suppl 2:S14–21. doi: 10.1007/PL00002934 11587045

[6] Chatterjee S Khunti K Davies MJ. Type 2 diabetes. Lancet (2017) 389:2239–51. doi: 10.1016/S0140-6736(17)30058-2 28190580

[7] Petersen MC Shulman GI. Mechanisms of insulin action and insulin resistance. Physiol Rev (2018) 98:2133–223. doi: 10.1152/physrev.00063.2017 PMC6170977 30067154

[8] American Diabetes Association. (2) Classification and diagnosis of diabetes. Diabetes Care (2015) 38:Suppl:S8–s16. 10.2337/dc15-S005 25537714

[9] Matthews DR Hosker JP Rudenski AS Naylor BA Treacher DF Turner RC. Homeostasis model assessment: Insulin resistance and beta-cell function from fasting plasma glucose and insulin concentrations in man. Diabetologia (1985) 28:412–9. doi: 10.1007/BF00280883 3899825

[10] Kolb H Martin S. Environmental/lifestyle factors in the pathogenesis and prevention of type 2 diabetes. BMC Med (2017) 15:131. doi: 10.1186/s12916-017-0901-x 28720102 PMC5516328

[11] Sladek R Rocheleau G Rung J Dina C Shen L Serre D. A genome-wide association study identifies novel risk loci for type 2 diabetes. Nature (2007) 445:881–5. doi: 10.1038/nature05616 17293876

[12] Zeggini E Weedon MN Lindgren CM Frayling TM Elliott KS Lango H. Replication of genome-wide association signals in UK samples reveals risk loci for type 2 diabetes. Science (2007) 316:1336–41. doi: 10.1126/science.1142364 PMC3772310 17463249

[13] Schulz MC Sargis RM. Inappropriately sweet: Environmental endocrine-disrupting chemicals and the diabetes pandemic. Adv Pharmacol (2021) 92:419–56. doi: 10.1016/bs.apha.2021.04.002 PMC8714029 34452693

[14] Margolis R Sant KE. Associations between exposures to perfluoroalkyl substances and diabetes, hyperglycemia, or insulin resistance: A scoping review. J Xenobiot (2021) 11:115–29. doi: 10.3390/jox11030008 PMC8482218 34564296

[15] Qi W Clark JM Timme-Laragy AR Park Y. Per- and polyfluoroalkyl substances and obesity, type 2 diabetes and non-alcoholic fatty liver disease: A review of epidemiologic findings. Toxicol Environ Chem (2020) 102:1–36. doi: 10.1080/02772248.2020.1763997 33304027 PMC7723340

[16] Buck RC Franklin J Berger U Conder JM Cousins IT de Voogt P. Perfluoroalkyl and polyfluoroalkyl substances in the environment: terminology, classification, and origins. Integr Environ Assess Manag (2011) 7:513–41. doi: 10.1002/ieam.258 PMC3214619 21793199

[17] Glüge J Scheringer M Cousins IT DeWitt JC Goldenman G Herzke D. An overview of the uses of per- and polyfluoroalkyl substances (PFAS). Environ Sci Process Impacts (2020) 22:2345–73. doi: 10.1039/D0EM00291G PMC7784712 33125022

[18] Prevedouros K Cousins IT Buck RC Korzeniowski SH. Sources, fate and transport of perfluorocarboxylates. Environ Sci Technol (2006) 40:32–44. doi: 10.1021/es0512475 16433330

[19] Trier X Granby K Christensen JH. Polyfluorinated surfactants (PFS) in paper and board coatings for food packaging. Environ Sci Pollut Res Int (2011) 18:1108–20. doi: 10.1007/s11356-010-0439-3 21327544

[20] Nickerson A Maizel AC Kulkarni PR Adamson DT Kornuc JJ Higgins CP. Enhanced extraction of AFFF-associated PFASs from source zone soils. Environ Sci Technol (2020) 54:4952–62. doi: 10.1021/acs.est.0c00792 32200626

[21] Meegoda JN Kewalramani JA Li B Marsh RW. A review of the applications, environmental release, and remediation technologies of per- and polyfluoroalkyl substances. Int J Environ Res Public Health (2020) 17(21):8117–43. doi: 10.3390/ijerph17218117 PMC7663283 33153160

[22] Buck RC Korzeniowski SH Laganis E Adamsky F. Identification and classification of commercially relevant per- and poly-fluoroalkyl substances (PFAS). Integr Environ Assess Manag (2021) 17:1045–55. doi: 10.1002/ieam.4450 PMC9292543 33991049

[23] (OECD). Ofec-oad: Toward a new comprehensive global database of per- and polyfluoroalkyl substances (Pfass): Summary report on updating the oecd 2007 list of per- and polyfluoroalkyl substances (Pfass) (2018). Available at: https://wwwoecdorg/officialdocuments/publicdisplaydocumentpdf/?cote=ENV-JM-MONO(2018)7&doclanguage=en.

[24] Zhang Z Sarkar D Biswas JK Datta R. Biodegradation of per- and polyfluoroalkyl substances (PFAS): A review. Bioresour Technol (2022) 344:126223. doi: 10.1016/j.biortech.2021.126223 34756980

[25] Brusseau ML Anderson RH Guo B. PFAS concentrations in soils: Background levels versus contaminated sites. Sci Total Environ (2020) 740:140017. doi: 10.1016/j.scitotenv.2020.140017 32927568 PMC7654437

[26] Domingo JL Nadal M. Human exposure to per- and polyfluoroalkyl substances (PFAS) through drinking water: A review of the recent scientific literature. Environ Res (2019) 177:108648. doi: 10.1016/j.envres.2019.108648 31421451

[27] Domingo JL Nadal M. Per- and polyfluoroalkyl substances (PFASs) in food and human dietary intake: A review of the recent scientific literature. J Agric Food Chem (2017) 65:533–43. doi: 10.1021/acs.jafc.6b04683 28052194

[28] Clara M Scheffknecht C Scharf S Weiss S Gans O. Emissions of perfluorinated alkylated substances (PFAS) from point sources–identification of relevant branches. Water Sci Technol (2008) 58:59–66. doi: 10.2166/wst.2008.641 18653937

[29] Calafat AM Wong LY Kuklenyik Z Reidy JA Needham LL. Polyfluoroalkyl chemicals in the U.S. population: Data from the national health and nutrition examination survey (NHANES) 2003-2004 and comparisons with NHANES 1999-2000. Environ Health Perspect (2007) 115:1596–602. doi: 10.1289/ehp.10598 PMC2072821 18007991

[30] Kato K Wong LY Jia LT Kuklenyik Z Calafat AM. Trends in exposure to polyfluoroalkyl chemicals in the U.S. population: 1999-2008. Environ Sci Technol (2011) 45:8037–45. doi: 10.1021/es1043613 21469664

[31] (CDC). CfDCaP: NHANES 2003-2004 public data general release file documentation (2005). Available at: https://wwwcdcgov/nchs/data/nhanes/nhanes_03_04/general_data_release_doc_03-04pdf.

[32] Everett CJ Frithsen I Player M. Relationship of polychlorinated biphenyls with type 2 diabetes and hypertension. J Environ Monit (2011) 13:241–51. doi: 10.1039/C0EM00400F 21127808

[33] Hopf NB Ruder AM Succop P. Background levels of polychlorinated biphenyls in the U. S. popul Sci Total Environ (2009) 407:6109–19. doi: 10.1016/j.scitotenv.2009.08.035 19773016

[34] Grandjean P Clapp R. Perfluorinated alkyl substances: Emerging insights into health risks. New Solut (2015) 25:147–63. doi: 10.1177/1048291115590506 PMC6172956 26084549

[35] Sunderland EM Hu XC Dassuncao C Tokranov AK Wagner CC Allen JG. A review of the pathways of human exposure to poly- and perfluoroalkyl substances (PFASs) and present understanding of health effects. J Expo Sci Environ Epidemiol (2019) 29:131–47. doi: 10.1038/s41370-018-0094-1 PMC6380916 30470793

[36] DeWitt JC Peden-Adams MM Keller JM Germolec DR. Immunotoxicity of perfluorinated compounds: recent developments. Toxicol Pathol (2012) 40:300–11. doi: 10.1177/0192623311428473 22109712

[37] Meneguzzi A Fava C Castelli M Minuz P. Exposure to perfluoroalkyl chemicals and cardiovascular disease: Experimental and epidemiological evidence. Front Endocrinol (Lausanne) (2021) 12:706352. doi: 10.3389/fendo.2021.706352 34305819 PMC8298860

[38] Rappazzo KM Coffman E Hines EP. Exposure to perfluorinated alkyl substances and health outcomes in children: A systematic review of the epidemiologic literature. Int J Environ Res Public Health (2017) 14(7):691–713. doi: 10.3390/ijerph14070691 PMC5551129 28654008

[39] Rickard BP Rizvi I Fenton SE. Per- and poly-fluoroalkyl substances (PFAS) and female reproductive outcomes: PFAS elimination, endocrine-mediated effects, and disease. Toxicology (2022) 465:153031. doi: 10.1016/j.tox.2021.153031 34774661 PMC8743032

[40] Steenland K Winquist A. PFAS and cancer, a scoping review of the epidemiologic evidence. Environ Res (2021) 194:110690. doi: 10.1016/j.envres.2020.110690 33385391 PMC7946751

[41] Domazet SL Grøntved A Timmermann AG Nielsen F Jensen TK. Longitudinal associations of exposure to perfluoroalkylated substances in childhood and adolescence and indicators of adiposity and glucose metabolism 6 and 12 years later: The European youth heart study. Diabetes Care (2016) 39:1745–51. doi: 10.2337/dc16-0269 27489335

[42] Kim JH Park HY Jeon JD Kho Y Kim SK Park MS. The modifying effect of vitamin c on the association between perfluorinated compounds and insulin resistance in the Korean elderly: a double-blind, randomized, placebo-controlled crossover trial. Eur J Nutr (2016) 55:1011–20. doi: 10.1007/s00394-015-0915-0 25939797

[43] Cardenas A Gold DR Hauser R Kleinman KP Hivert MF Calafat AM. Plasma concentrations of per- and polyfluoroalkyl substances at baseline and associations with glycemic indicators and diabetes incidence among high-risk adults in the diabetes prevention program trial. Environ Health Perspect (2017) 125:107001. doi: 10.1289/EHP1612 28974480 PMC5933403

[44] Duan Y Sun H Yao Y Meng Y Li Y. Distribution of novel and legacy per-/polyfluoroalkyl substances in serum and its associations with two glycemic biomarkers among Chinese adult men and women with normal blood glucose levels. Environ Int (2020) 134:105295. doi: 10.1016/j.envint.2019.105295 31726357

[45] Alderete TL Jin R Walker DI Valvi D Chen Z Jones DP. Perfluoroalkyl substances, metabolomic profiling, and alterations in glucose homeostasis among overweight and obese Hispanic children: A proof-of-concept analysis. Environ Int (2019) 126:445–53. doi: 10.1016/j.envint.2019.02.047 PMC6555482 30844580

[46] Zeeshan M Zhang YT Yu S Huang WZ Zhou Y Vinothkumar R. Exposure to isomers of per- and polyfluoroalkyl substances increases the risk of diabetes and impairs glucose-homeostasis in Chinese adults: Isomers of C8 health project. Chemosphere (2021) 278:130486. doi: 10.1016/j.chemosphere.2021.130486 34126693

[47] Valvi D Højlund K Coull BA Nielsen F Weihe P Grandjean P. Life-course exposure to perfluoroalkyl substances in relation to markers of glucose homeostasis in early adulthood. J Clin Endocrinol Metab (2021) 106:2495–504. doi: 10.1210/clinem/dgab267 PMC8277200 33890111

[48] Liu HS Wen LL Chu PL Lin CY. Association among total serum isomers of perfluorinated chemicals, glucose homeostasis, lipid profiles, serum protein and metabolic syndrome in adults: NHANES, 2013-2014. Environ pollut (2018) 232:73–9. doi: 10.1016/j.envpol.2017.09.019 28923343

[49] Nelson JW Hatch EE Webster TF. Exposure to polyfluoroalkyl chemicals and cholesterol, body weight, and insulin resistance in the general U.S. population. Environ Health Perspect (2010) 118:197–202. doi: 10.1289/ehp.0901165 20123614 PMC2831917

[50] Fisher M Arbuckle TE Wade M Haines DA. Do perfluoroalkyl substances affect metabolic function and plasma lipids?–Analysis of the 2007-2009, Canadian health measures survey (CHMS) cycle 1. Environ Res (2013) 121:95–103. doi: 10.1016/j.envres.2012.11.006 23266098

[51] Fleisch AF Rifas-Shiman SL Mora AM Calafat AM Ye X Luttmann-Gibson H. Early-life exposure to perfluoroalkyl substances and childhood metabolic function. Environ Health Perspect (2017) 125:481–7. doi: 10.1289/EHP303 PMC5332186 27586368

[52] Fassler CS Pinney SE Xie C Biro FM Pinney SM. Complex relationships between perfluorooctanoate, body mass index, insulin resistance and serum lipids in young girls. Environ Res (2019) 176:108558. doi: 10.1016/j.envres.2019.108558 31271921 PMC6739842

[53] Lundin JI Alexander BH Olsen GW Church TR. Ammonium perfluorooctanoate production and occupational mortality. Epidemiology (2009) 20:921–8. doi: 10.1097/EDE.0b013e3181b5f395 19797969

[54] Sun Q Zong G Valvi D Nielsen F Coull B Grandjean P. Plasma concentrations of perfluoroalkyl substances and risk of type 2 diabetes: A prospective investigation among U.S. women. Environ Health Perspect (2018) 126:037001. doi: 10.1289/EHP2619 29498927 PMC6071816

[55] He X Liu Y Xu B Gu L Tang W. PFOA is associated with diabetes and metabolic alteration in US men: National health and nutrition examination survey 2003-2012. Sci Total Environ (2018) 625:566–74. doi: 10.1016/j.scitotenv.2017.12.186 29291571

[56] Girardi P Merler E. A mortality study on male subjects exposed to polyfluoroalkyl acids with high internal dose of perfluorooctanoic acid. Environ Res (2019) 179:108743. doi: 10.1016/j.envres.2019.108743 31542491

[57] Cardenas A Hivert MF Gold DR Hauser R Kleinman KP Lin PD. Associations of perfluoroalkyl and polyfluoroalkyl substances with incident diabetes and microvascular disease. Diabetes Care (2019) 42:1824–32. doi: 10.2337/dc18-2254 PMC6702604 31296647

[58] Donat-Vargas C Bergdahl IA Tornevi A Wennberg M Sommar J Kiviranta H. Perfluoroalkyl substances and risk of type II diabetes: A prospective nested case-control study. Environ Int (2019) 123:390–8. doi: 10.1016/j.envint.2018.12.026 30622063

[59] Karnes C Winquist A Steenland K. Incidence of type II diabetes in a cohort with substantial exposure to perfluorooctanoic acid. Environ Res (2014) 128:78–83. doi: 10.1016/j.envres.2013.11.003 24299613

[60] MacNeil J Steenland NK Shankar A Ducatman A. A cross-sectional analysis of type II diabetes in a community with exposure to perfluorooctanoic acid (PFOA). Environ Res (2009) 109:997–1003. doi: 10.1016/j.envres.2009.08.002 19740462

[61] Conway B Innes KE Long D. Perfluoroalkyl substances and beta cell deficient diabetes. J Diabetes Complications (2016) 30:993–8. doi: 10.1016/j.jdiacomp.2016.05.001 PMC5556924 27311784

[62] Charles D Berg V Nøst TH Huber S Sandanger TM Rylander C. Pre- and post-diagnostic blood profiles of perfluoroalkyl acids in type 2 diabetes mellitus cases and controls. Environ Int (2020) 145:106095. doi: 10.1016/j.envint.2020.106095 32919259

[63] Schillemans T Shi L Donat-Vargas C Hanhineva K Tornevi A Johansson I. Plasma metabolites associated with exposure to perfluoroalkyl substances and risk of type 2 diabetes - a nested case-control study. Environ Int (2021) 146:106180. doi: 10.1016/j.envint.2020.106180 33113464

[64] Han X Meng L Zhang G Li Y Shi Y Zhang Q. Exposure to novel and legacy per- and polyfluoroalkyl substances (PFASs) and associations with type 2 diabetes: A case-control study in East China. Environ Int (2021) 156:106637. doi: 10.1016/j.envint.2021.106637 33993001

[65] Mancini FR Rajaobelina K Praud D Dow C Antignac JP Kvaskoff M. Nonlinear associations between dietary exposures to perfluorooctanoic acid (PFOA) or perfluorooctane sulfonate (PFOS) and type 2 diabetes risk in women: Findings from the E3N cohort study. Int J Hyg Environ Health (2018) 221:1054–60. doi: 10.1016/j.ijheh.2018.07.007 30056006

[66] Duan Y Sun H Yao Y Li Y Meng Y Lu Y. Serum concentrations of per-/polyfluoroalkyl substances and risk of type 2 diabetes: A case-control study. Sci Total Environ (2021) 787:147476. doi: 10.1016/j.scitotenv.2021.147476 33992947

[67] Lin PD Cardenas A Hauser R Gold DR Kleinman KP Hivert MF. Per- and polyfluoroalkyl substances and kidney function: Follow-up results from the diabetes prevention program trial. Environ Int (2021) 148:106375. doi: 10.1016/j.envint.2020.106375 33482440 PMC7929640

[68] Park SK Wang X Ding N Karvonen-Gutierrez CA Calafat AM Herman WH. Per- and polyfluoroalkyl substances and incident diabetes in midlife women: The study of women's health across the nation (SWAN). Diabetologia (2022) 65(7):1157–68. doi: 10.1007/s00125-022-05695-5 PMC9177697 35399113

[69] Yan S Zhang H Zheng F Sheng N Guo X Dai J. Perfluorooctanoic acid exposure for 28 days affects glucose homeostasis and induces insulin hypersensitivity in mice. Sci Rep (2015) 5:11029. doi: 10.1038/srep11029 26066376 PMC4464286

[70] Zheng F Sheng N Zhang H Yan S Zhang J Wang J. Perfluorooctanoic acid exposure disturbs glucose metabolism in mouse liver. Toxicol Appl Pharmacol (2017) 335:41–8. doi: 10.1016/j.taap.2017.09.019 28947262

[71] Qin W Ren X Zhao L Guo L. Exposure to perfluorooctane sulfonate reduced cell viability and insulin release capacity of β cells. J Environ Sci (China) (2022) 115:162–72. doi: 10.1016/j.jes.2021.07.004 34969446

[72] Duan X Sun W Sun H Zhang L. Perfluorooctane sulfonate continual exposure impairs glucose-stimulated insulin secretion *via* SIRT1-induced upregulation of UCP2 expression. Environ Pollut (2021) 278:116840. doi: 10.1016/j.envpol.2021.116840 33689947

[73] Hoyeck MP Matteo G MacFarlane EM Perera I Bruin JE. Persistent organic pollutants and β-cell toxicity: A comprehensive review. Am J Physiol Endocrinol Metab (2022) 322:E383–e413. doi: 10.1152/ajpendo.00358.2021 35156417 PMC9394781

